# Supplementary material for: Global gene expression analyses of the alkamide-producing plant Heliopsis longipes supports a polyketide synthase-mediated biosynthesis pathway
Source: PeerJ. 2020 Sep 25;8:e10074. doi: 10.7717/peerj.10074 (PMC7521342; doi:10.7717/peerj.10074)
Supplement: Supplemental Information 1 [file peerj-08-10074-s001.docx]

**Table S1:**

**Accession numbers and organism of origin of the sequences used for the phylogenetic analysis of KS domains.**

| **Accession** | **Organism** | **Accession** | | **Organism** |
| --- | --- | --- | --- | --- |
| NP 199441.1 | *Arabidopsis thaliana* | WP 093651808.1 | *Streptomyces wuyuanensis* | |
| AGC65509.1 | *Carthamus tinctorius* | AIL50179.1 | *Streptomyces aureus* | |
| KVG91898.1 | *Cynara cardunculus* | CAK50776.1 | *Streptomyces argillaceus* | |
| KVI00884.1 | *Cynara cardunculus* | AAV48829.2 | *Streptomyces spiramyceticus* | |
| XP 022025436.1 | *Helianthus annuus* | AAB36562.1 | *Streptomyces venezuelae* | |
| OJV07714.1 | *Chlamydiales bacterium* | NP 199441.1 | *Arabidopsis thaliana* | |
| CRX38508.1 | *Estrella lausannensis* | AAB61310. | *Allium ampeloprasum* | |
| WP_042103505.1 | *Parachlamydiaceae bacterium* | ABE73469.1 | *Elaeis guineensis* | |
| WP 068470483.1 | *Parachlamydia sp.* | EOY27735.1 | *Theobroma cacao* | |
| WP_032125192.1 | *Chlamydia sp.* | XP 002863412.1 | *Arabidopsis lyrata* | |
| WP_059060254.1 | *Protochlamydia naegleriophila* | CAA80452.1 | *Spinacia oleracea* | |
| WP_039359465.1 | *Protochlamydia amoebophila* | EYB32591.1 | *Fusarium graminearum* | |
| WP_075883222.1 | *Protochlamydia* | AJE53317.1 | *Paenibacillus polymyxa* | |
| NP 081971.1 | *Mus musculus* | AEZ53952.1 | *Streptomyces albus* | |
| NP_001093978.1 | *Rattus norvegicus* | AGL16670.1 | *Actinoplanes sp.* | |
| OBS66480.1 | *Neotoma lepida* | AHH94845.1 | *Kutzneria albida* | |
| XP_005081045.1 | *Mesocricetus auratus* | WP_044568899.1 | *Streptomyces iranensis* | |
| XP_021534485.1 | *Neomonachus schauinslandi* | WP_043437269.1 | *Streptomyces nodosus* | |
| XP_012290981.1 | *Aotus nancymaae* | WP_015801519.1 | *Actinosynnema mirum* | |
| XP_008057440.1 | *Carlito syrichta* | ABP55210.1 | *Salinispora tropica* | |
| pdb 2C9H | *mitochondrial Chain A* | ADI03792.1 | *Streptomyces bingchenggensis* | |
| NP 060367.1 | *Homo sapiens* | WP_048894517.1 | *Streptomyces avermitilis* | |
| pdb 2IWY | *Human Mitochondrial* | WP_062008639.1 | *Streptomyces hygroscopicus* | |
| WP 031486374.1 | *Streptomyces bicolor* | QDQ16835.1 | *Streptomyces sporocinereus* | |

*Data taken from NCBI (<https://www.ncbi.nlm.nih.gov>)
