# Supplementary material for: Global gene expression analyses of the alkamide-producing plant Heliopsis longipes supports a polyketide synthase-mediated biosynthesis pathway
Source: PeerJ. 2020 Sep 25;8:e10074. doi: 10.7717/peerj.10074 (PMC7521342; doi:10.7717/peerj.10074)
Supplement: Supplemental Information 2 [file peerj-08-10074-s002.docx]

**Table S2:**

**Primer pairs used to detect the expression levels of candidate genes by quantitative real-time PCR.**

| Gene | Left primer (F) sequence  (5´ 🡪 3´ direction) | Size (bp) | % GC | TM  (°C) | Right primer (R) sequence  (5´🡪 3´ direction) | Size (bp) | % GC | TM  (°C) |
| --- | --- | --- | --- | --- | --- | --- | --- | --- |
| **ACT** | CATTGTGCTCAGTGGTGGTTC | 21 | 52 | 65 | TCTGCTGGAAGGTGCTAAGTG | 21 | 52 | 64 |
| **KAS 4** | GTCGCACAAGCTGTTGAAAT | 20 | 45 | 58.9 | GCACTCATCCAAAAGGGATT | 20 | 45 | 59 |
| **AT** | GCCATAACATGTGCATCTCC | 20 | 50 | 59 | GCTTTTCGTAGGCTTGTTCC | 20 | 50 | 59 |
| **AT** | CAGGCCATATCATTTGCATC | 20 | 45 | 58.9 | GCTTTTCGTAGGCTTGTTCC | 20 | 50 | 59 |
| **ACP** | CCTGTTGTTTCCACCTTCCT | 20 | 50 | 59 | AATTAGAGGCCCGGGTAAGT | 20 | 50 | 59 |
| **OR 3** | TCTTGGATCAGCTTGAGTGG | 20 | 50 | 59 | TGTGGTGTAACTCCGTCGAT | 20 | 50 | 59 |
| **OR 4** | GCTTGCAAAGTTCTCAACCA | 20 | 45 | 59 | CTGGAGTGGTCATTGTGGAC | 20 | 55 | 58.9 |
| **OR 5** | GCCTCCAAAACGGATGTAAT | 20 | 45 | 58.9 | TGGAACGGTTGGTTGAGTTA | 20 | 45 | 59 |
| **DH 1** | GTTTATGCGTGCAATCTGCT | 20 | 45 | 58.9 | TGGCATGTTTCATCGATTTC | 20 | 40 | 59.5 |
| **DH 2** | TGACAGTTTTGGATGGTGGT | 20 | 45 | 58.8 | AGAGAGAGATGGCACCGACT | 20 | 55 | 59 |
| **DH 3** | GCTCGGTTCTCTTTTGGAAC | 20 | 50 | 58.9 | CATGAGATCGCCTTCGATTA | 20 | 45 | 58.8 |
| **TE 1** | CGATGGATTTGCCACTACAC | 20 | 50 | 59 | CCAGGTCTCAACTTCGATCA | 20 | 50 | 58.8 |
| **TE 2** | TCACCCACTCCCTTATCTCC | 20 | 55 | 58.9 | AAGCACAATTGGTGGTGAAA | 20 | 40 | 59 |

**F**: forward, **R**: reverse, **bp**: base pairs, **% GC**: guanine-cytosine percentage, **TM**: melting temperature, **ACT**: actin, **KS**: ketosynthase, **AT**: acyl transferase, **ACP**: acyl carrier protein, **OR**: oxide reductase, **DH**: dehydratase, **TE:** thioesterase.
